# Selectivity/Specificity Improvement Strategies in Surface-Enhanced Raman Spectroscopy Analysis

**DOI:** 10.3390/s17112689

**Published:** 2017-11-21

**Authors:** Feng Wang, Shiyu Cao, Ruxia Yan, Zewei Wang, Dan Wang, Haifeng Yang

**Affiliations:** The Education Ministry Key Lab of Resource Chemistry, Shanghai Key Laboratory of Rare Earth Functional Materials, Shanghai Municipal Education Committee Key Laboratory of Molecular Imaging Probes and Sensors, and Department of Chemistry, Shanghai Normal University, Shanghai 200234, China; 1000422898@smail.shnu.edu.cn (S.C.); 15800386391@163.com (R.Y.); wangzewei1226@163.com (Z.W.); 18817289115@163.com (D.W.)

**Keywords:** surface-enhanced Raman spectroscopy, specificity, selectivity, chemical reaction, antibody, aptamer, molecularly imprinted polymers, microfluidics

## Abstract

Surface-enhanced Raman spectroscopy (SERS) is a powerful technique for the discrimination, identification, and potential quantification of certain compounds/organisms. However, its real application is challenging due to the multiple interference from the complicated detection matrix. Therefore, selective/specific detection is crucial for the real application of SERS technique. We summarize in this review five selective/specific detection techniques (chemical reaction, antibody, aptamer, molecularly imprinted polymers and microfluidics), which can be applied for the rapid and reliable selective/specific detection when coupled with SERS technique.

## 1. Introduction

Sensitive detection of target analytes among myriads of other components is of vital importance for sample analysis, process monitoring and mechanism study. Nowadays the “gold standard” methods for this aim are chromatography-related techniques, such as high performance liquid chromatography tandem mass spectroscopy (HPLC-MS) and gas chromatography tandem mass spectrometry (GC-MS) [1]. However, these methods invariably need expensive equipment and highly trained technicians. In addition, these techniques are neither cost-effective nor time-saving in high-throughput screening of large amounts of samples. Therefore, spectroscopic techniques (especially fluorescence, ultraviolet-visible and Raman spectroscopy) have been introduced to overcome such drawbacks.

Raman scattering is the inelastic scattering of photons which can reflect the vibrational or rotational modes of the interacting molecules [2]. Raman spectroscopy has many unique features compared to other spectroscopic techniques. For example, the frequency shifts of Raman scattering can provide “fingerprint” information of analyte’s chemical structure. Besides, Raman peaks are very narrow, which facilitate multicomponent analysis and quantification. Additionally, water has negligible influence on the final Raman spectrum, which is an advantage to apply Raman study in aqueous or physiological environment. However, only a very small fraction of the scattered photons (about 1 out of 10 million possibility) are scattered inelastically, the cross section of Raman scattering is also very small (about 14 orders lower than that of fluorescence emission; the cross section of two interacting particles is the area transverse to their relative motion in which they will meet and scatter from each other, the scattering cross section of particles interacting through electromagnetic force is generally larger than their geometric size). Therefore, the Raman signal is very weak and can hardly be used for trace detection.

In 1973, Fleischmann et al. found that the Raman signal of pyridine adsorbed on a roughened silver electrode can be significantly enhanced [3]. This phenomenon was later termed SERS, which describes the unusual Raman signal enhancement when molecules are adsorbed or are close to rough metal surfaces or nanostructures [4]. Two main mechanisms have been proposed to explain this SERS phenomenon. Electromagnetic enhancement theory believes that the incident light can excite electrons on the nanostructured metal surfaces to generate localized surface plasmon resonance (LSPR), such electromagnetic enhancement in an electromagnetic field induced by LSPR plays a leading role in SERS enhancement. In comparison, chemical enhancement (also called charge-transfer effect) mechanism can only apply on species that can form chemical bonds with metal surfaces. Due to the fact that SERS enhancement factor can reach as high as ~10^11^, which make even single molecule detection possible [5], SERS has already been widely accepted as a sensitive and high-throughput technique. 

However, issues still need to be addressed before exploiting SERS method for analyte diagnosis in multicomponent systems. The most serious problem is that SERS itself, as a spectroscopic technique, is good at structure characterization but poor in component separation. In a multicomponent system each component contributes to the final SERS spectrum, especially those that have large Raman scattering cross section or have strong interactions with SERS substrates. Though this problem can be alleviated by pretreatment methods such as extraction/centrifugation, the final SERS spectrum may still be influenced by homologues or compounds bearing similar properties with target analytes. Chemometric methods also can’t provide satisfying discrimination result in samples where the target analyte is among dozens of impurities. Therefore, in order to achieve selective/specific SERS detection, separation/specific recognition method is in demand before spectral collection. Either chemical or physical method can be applied in the separation/specific recognition process. Five reprehensive selective/specific detection techniques tandem SERS method (chemical reaction-SERS, antibody-SERS, aptamer-SERS, molecularly imprinted polymer-SERS and micro-fluidics-SERS) will be reviewed in this article.

## 2. Reaction-SERS Method

**Scheme 1:**
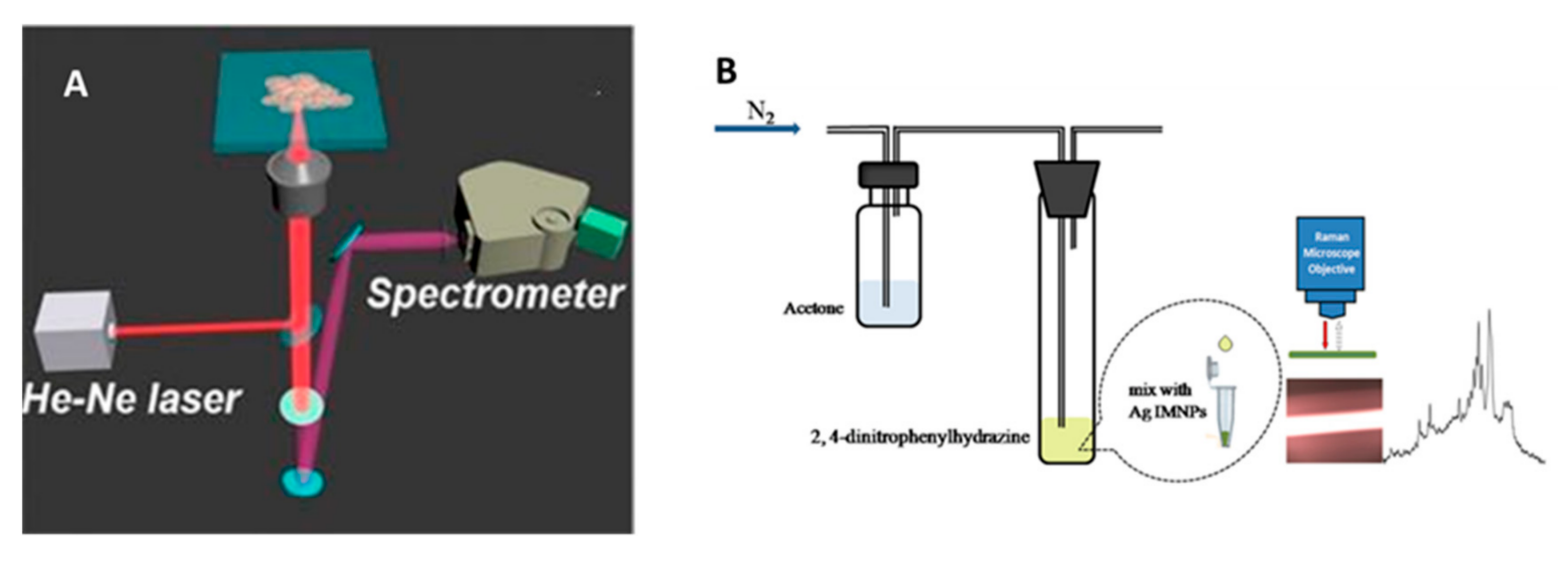
(**A**) Schematic diagram of Raman detection; (**B**) Schematic diagram of chemical reaction assisted SERS detection [17].

Analytes possessing little affinity to the SERS substrate or having small Raman cross sections are usually difficult to be detected by SERS technology [6]. In addition, gaseous analytes are also difficult to be analyzed by SERS because of their volatility and low molecular weight [7], which result in poor sensitivity, selectivity and accuracy. Besides, some unstable analytes also need to be converted into stable products before the detection [8]. In such cases, chemical reactions are introduced before SERS detection. The schematic diagram of Raman detection and chemical reaction-SERS can be seen in Scheme 1. Though chemical reactions have already been used in conjunction with other analytical methods such as LC-MS [9,10], HPLC [11,12], HPLC-MS [13], fluorescence spectrometry [14,15], colorimetric methods [16] and so on to improve the sensitivity or selectivity, these methods are either time consuming or cost efficient. 

In general, compounds with conjugated structures have strong SERS response. Increasing the Raman scattering cross section can improve the SERS signal but reducing the Raman scattering cross section has the opposite effect. Additionally, there are some rules that should be followed in chemical reaction—SERS. Firstly, the compound should have a weak fluorescent background in order not to interfere SERS measurement. Simultaneously, it should have groups like –SH or –NH_2_ which can firmly combine with the SERS substrate. Thirdly, reagents should possess obvious spectra variations before and after chemical reactions. It can be classified into three cases, including “signal on”, “signal off” and “signal change” in which both reactants and products have strong SERS signals. Finally, the reaction reagent should have a good selectivity to the analyte which can be analyzed by the fingerprint spectrum. To sum up, chemical reactions—SERS can be divided into three categories.

### 2.1. Improving Analyte Affinity with SERS Substrate

**Figure 1:**
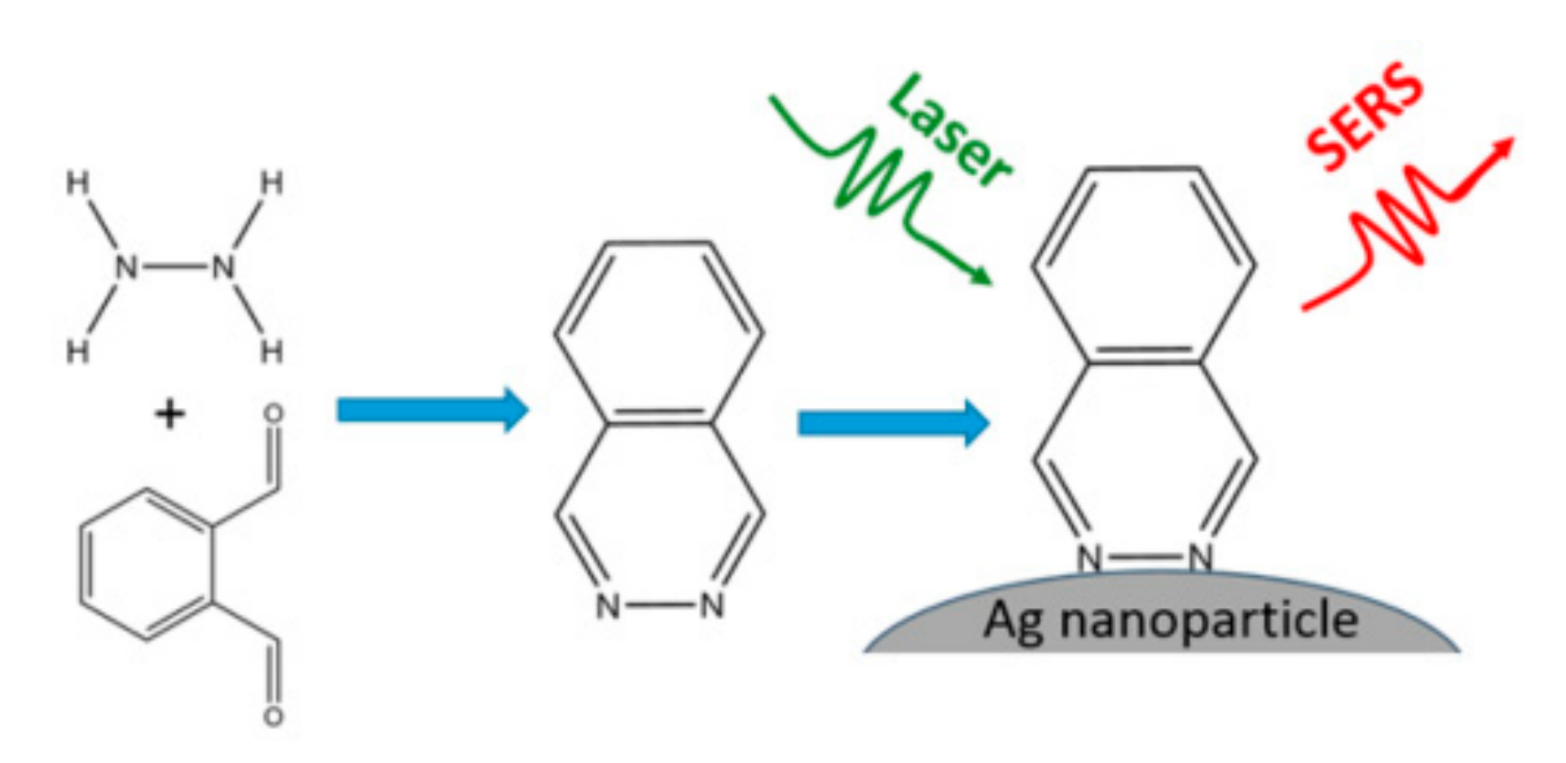
Scheme for SERS-based hydrazine detection derivatized with a phthaldialdehyde probe [6].

**Figure 2:**
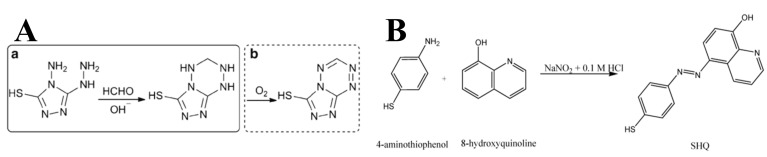
Derivatization reaction of HCHO and 4-amino-5-hydrazino-3-mercapto-1,2,4-triazole (AHMT) under alkaline conditions (**A**) [18]. Synthesis of p-([8-hydroxyquinoline]azo)benzenethiol (SHQ) (**B**) [19].

Many analytes having low affinity with gold or silver are not suitable for SERS detection. It can be solved by reacting with derivative reagents which can strongly bind to SERS substrates. Such derivative reagents normally bear thio or amino groups that can strongly bind with gold or silver SERS substrates. This method is flexible, easy to use and has high sensitivity and selectivity. However, the prerequisite of both analyte-reacting group and gold/silver binding group limits the choice of the derivative reagents. Camden et al. [6] reacted hydrazine with ortho-phthaldialdehyde to form phthalazine, which had better affinity toward the SERS substrate than hydrazine (Figure 1). The limit of detection (LOD, normally defined as signal to noise = 3) was 8.5 × 10^−11^ M, more sensitive than 6.3 × 10^−11^ M detected by GC/MS which was the best detection limit reported previously. Wang et al. [18] reported the derivatization of 4-amino-5-hydrazino-3-mercapto-1,2,4-triazole (AHMT) with HCHO (Figure 2A). SERS band at 832 cm^−1^ was then applied for HCHO quantification. This method is convenient, organic solvent free, low cost and can be used for on-site rapid detection of trace HCHO. Harris et al. [19] immobilized 8-hydroxyquinoline on the surface of the silver electrode by azo reaction with 4-aminothiophenol (Figure 2B) to coordinate with Cu^2+^. They observed that the tautomerization equilibrium could be controlled through the applied potential. 

### 2.2. Increasing the Raman Scattering Cross-Sectional Area

**Figure 3:**
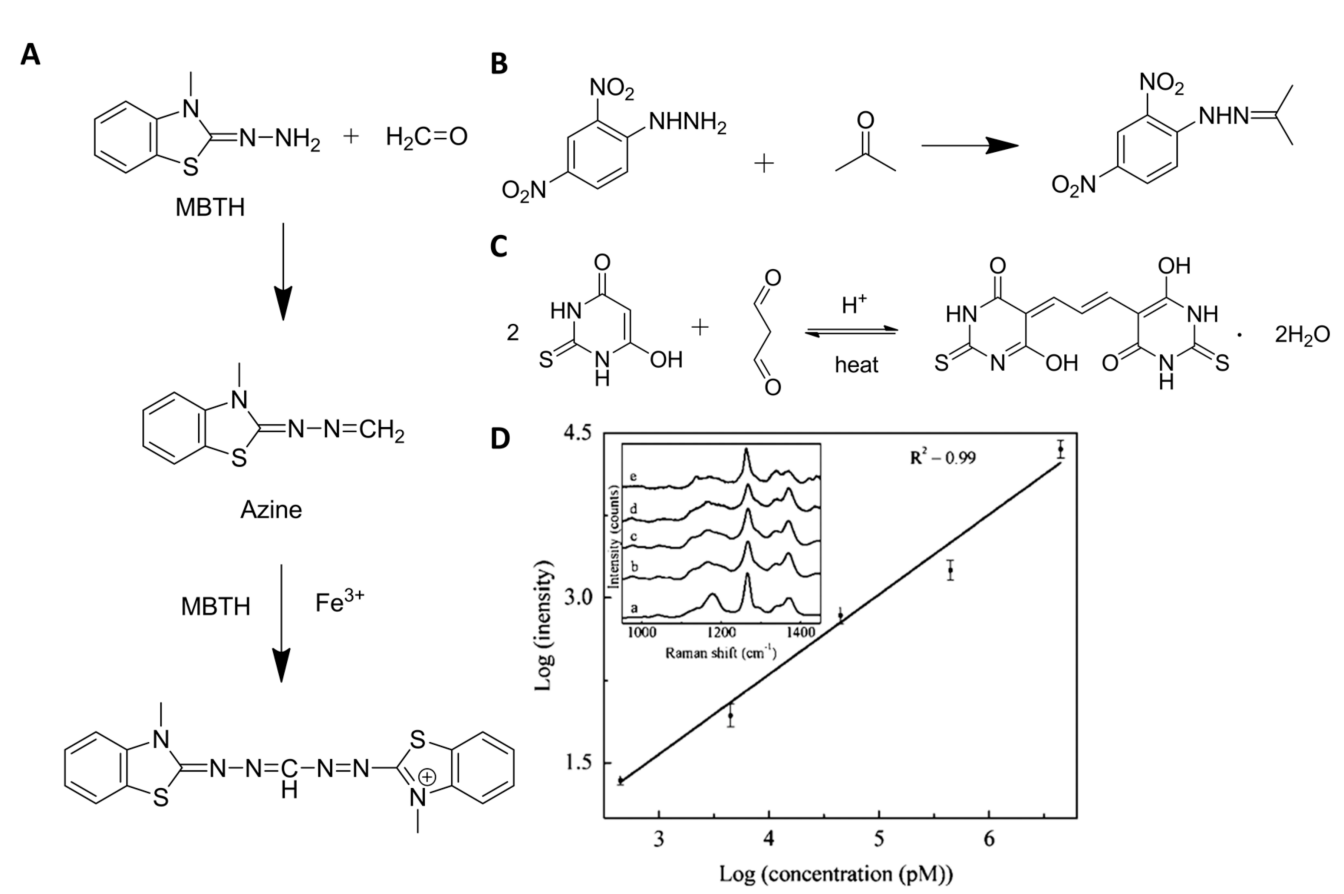
(**A**) Chemical derivatization reaction between MBTH hydrochloride and formaldehyde azine [20]; (**B**) Chemical derivatization reaction between acetone and 2,4-dinitrophenylhydrazine to form 2,4-dinitrophenyl hydrazine [17]; (**C**) Chemical derivatization reaction between thiobarbituric acid and malondialdehyde [21]; (**D**) Correlation between the SERS intensity at 1265 cm^−1^ of HPLC-purified TBA–MDA adduct and its concentration in picomolar, both in logarithm scales. Inset: representative SERS spectra of TBA–MDA with concentration of (a) 4.5 μM; (b) 0.45 μM; (c) 45 nM; (d) 4.5 nM; and (e) 0.45 nM [21].

Reactions that can increase the Raman scattering cross-sectional area of analytes are especially suitable of the SERS detection of small molecules. This chemical derivatization reaction can highly enlarge the SERS signal of analytes with simple structures which have no obvious Raman signal. Furthermore, it also can change the affinity of analytes to the SERS substrates, which can widely expand the application area of SERS technique. For example, Li et al. [20] used the derivative reagent of 3-Methyl-2-benzothiazolinone hydrazone (MBTH) hydrochloride monohydrate to detect trace formaldehyde in aquatic products by SERS (Figure 3A). Tian et al. [17] detected acetone via a derivatization reaction between 2,4-dinitrophenylhydrazine (2,4-DNPH) and acetone (Figure 3B). This method is fast, convenient, and economic to detect acetone in artificial urine and human urine samples. Pittman Jr. et al. [21] utilized thiobarbituric acid (TBA) to react with malondialdehyde (MDA) to monitor lipid peroxidation (Figure 3C). SERS detection limit of TBA–MDA was 0.45 nM (Figure 3D).

### 2.3. Reducing the Raman Scattering Cross-Sectional Area

Contrary to increasing the Raman scattering cross-sectional area, another reaction method that can be applied in SERS detection is to reduce the Raman scattering cross-sectional area of the analyte. For example, Li et al. [22] reported the reaction of bis[4,4′-dithiodiphenylazophenol] (DTDPAP) with sodium dithionite. This method is used to detect sodium dithionite and has fast response (5 min), low LOD (0.08 μM), and good selectivity. Whatever increasing or reducing the Raman scattering cross-sectional area, these two methods both have the disadvantages that it is a challenge to choose the reagents which possess obvious spectra variation before and after reactions.

Some special reactions were also developed to detect target analytes, especially gas targets by SERS. For example, Li et al. [7] used bromine-thiourea and OPA-NH_4_^+^ derivative reagents to derivatize with ethylene and SO_2_, respectively, which have low molecular weight and strong volatility. 

### 2.4. Application of Reaction-SERS Method

Reaction-SERS method has been widely used in many fields. As mentioned previously, reaction-SERS method is especially suitable for gas detection. For example, *p*-aminothiophenol (4-ATP) was used to detect gaseous aldehydes as biomarkers of lung cancer [23]. Compared with other gaseous molecule detection techniques such as GC-MS, ion mobility spectrometry, selected ion flow tube MS and chemical sensors, reaction-SERS method avoid time consuming pretreatments and can detect low concentration chemicals in air.

Reaction-SERS method was also applied in the detection of hazardous chemicals. Wu et al. [24] monitored the aldol condensation of surface-bound 4-(methylthio)benzaldehyde with free acetone and 4-nitrothiophenol was used as an internal standard (IS) to improve the SERS quantification. In another case, nitric acid and sulfuric acid produced by NO_2_ and SO_2_ were reacted with ammonium hydroxide to produce the corresponding ammonium salt without loss of chemical discrimination [25]. The LOD by this method was about 4 orders lower compared with that of LC method.

Reaction-SERS method was also applied in mechanism study. Cu^2+^ mediated *o*-phenylene-diamine (OPD) oxidation [26] on the surface of AuNPs was monitored by SERS. Hu et al. [27] reported that palladacycle-mediated carbonylation with carbon monoxide (CO) as a C1 source could be monitored in situ by SERS. In another case, the mechanism of two *p*-aminothiophenol molecules [28] oxidatively transforming into 4,4′-dimercaptoazobenzene (DMAB) by azo reaction was monitored by SERS. What’s more, chemical reactions could also be used to remove signal interferences from impurities that have strong SERS signal. We used an azo coupling pretreatment to remove the interference of urea and detected adenosine directly from urine samples [29].

Last but not the least, Reaction-SERS was also widely used in cells. Tian et al. [30] reported that the reactions of 4-mercaptophenol (4-MP) with ClO^−^ and glutathione (GSH) on the surface of gold flowers (AuF/MP) could be monitored by SERS. This SERS system showed high selectivity for the detection of ClO^−^ and GSH even in the presence of other reactive oxygen species and amino acids. NO in human body [31] could be detected by SERS through catalyzing OPD molecules to benzotriazole. Although colorimetry, electrochemical detection, mass spectrometry and fluorescence probes approaches had been developed to better elucidate the pathophysiological roles NO plays. However, most NO detection methods were not suitable for in situ detection in living cells [31] and fluorescence approach was limited by photobleaching and phototoxicity induced by the excitation light [32]. 

Chemical reactions in conjunction with SERS method can improve the selectivity and sensitivity of some analytes. Representative studies of reaction-SERS methods are summarized in Table 1. However, this method is difficult to distinguish homologues due to their similar functional groups and spectral information [33]. Another issue is the limited reaction types which can be used in reaction-SERS system. In addition, low reproducibility [34] was reported in some cases due to the uncontrolled aggregation of plasmonic NPs during reaction. Therefore, suitable SERS substrates that are more stable during reactions need to be developed.

## 3. Antibody-SERS

Together with the specificity of antibody-antigen interactions and the sensitivity of SERS, antibody-SERS method has already been applied in the selective detection of analytes in multicomponent systems, especially in physiological environments. Compared with other detection technique (fluorescence (FL), UV-Vis and chemoluminescence) in immunoassay, SERS has its unique advantages. The Raman signal of different Raman tags can be acquired utilizing one laser source. What’s more, peaks in SERS spectrum are very narrow compared to that of FL and UV-Vis, which enables the multianalyte detection at the same time. Furthermore, SERS signal is less vulnerable to photobleaching, quenching, and environmental factor variations (temperature, viscosity, pH value, etc.).

**Figure 4:**
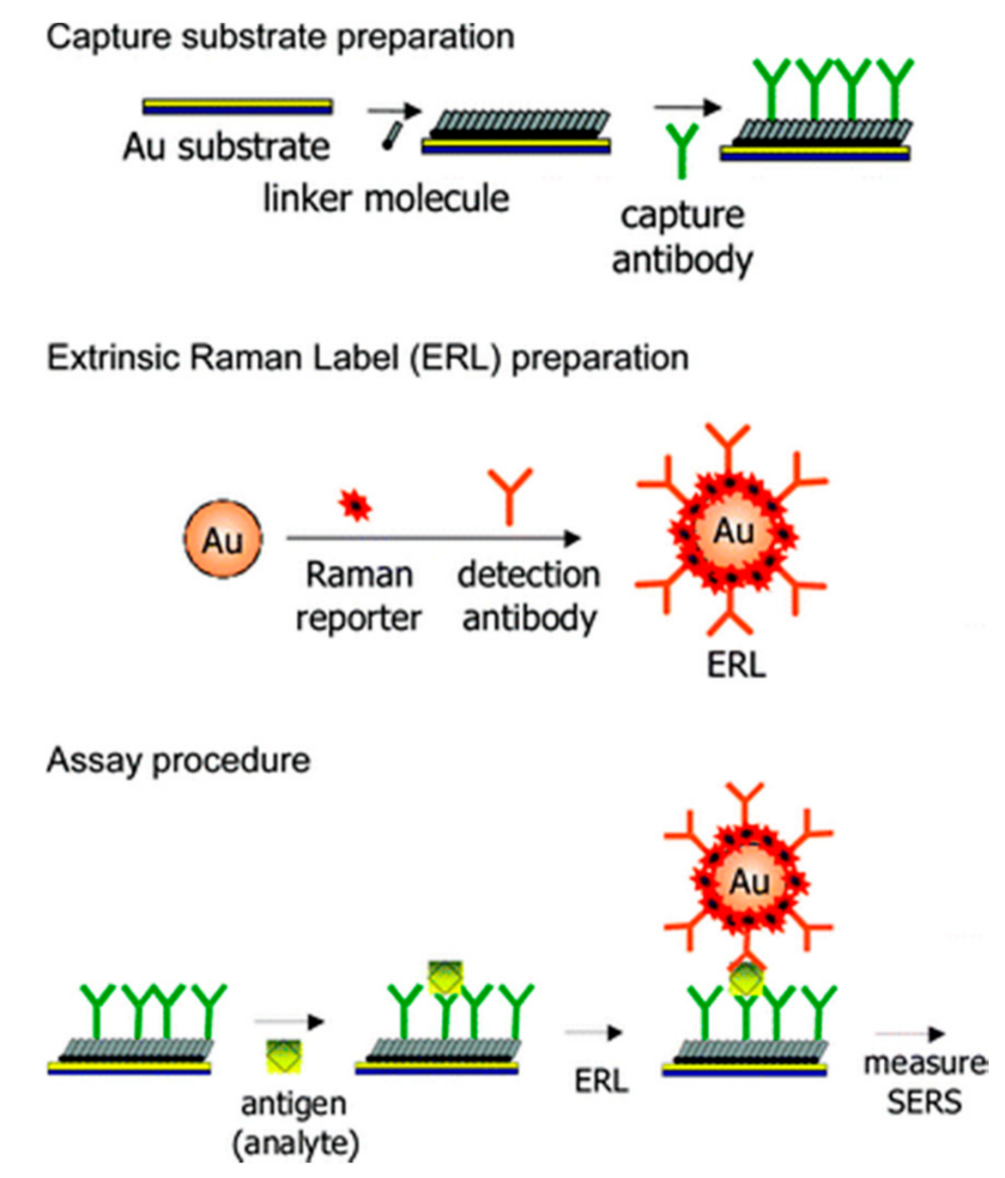
SERS-based immunoassay platform [35]. Antibodies were fixed on a platform to capture antigen, Ag or Au NPs conjugated with both Raman tags and detection antibodies can then be captured for SERS detection.

Rohr et al. reported the first Antibody-SERS study in 1989 to detect thyroid stimulating hormone (THS) utilizing sandwich immunoassay [36]. Figure 4 shows a representative indirect antibody-SERS sandwich-structure. The capturing antibodies were immobilized on the surface of a SERS substrate. Antigens from the sample solutions were then captured by these immobilized antibodies. These antigens can then capture Ag or Au NPs which were conjugated with both Raman tags and antibodies. The amount of antigen can then be indirectly acquired by measuring the SERS intensities of Raman tags.

Nowadays the antibody-SERS researches are mainly focus on how to increase sensitivity and decrease non-specific adsorption. One way to increase the sensitivity is to introduce more SERS tags. Porter et al. designed bifunctional SERS tags which contain a disulfide group for anchoring on nanoparticle surface and a succinimide group for antibody coupling [37]. This can minimize the separation steps and maximize the number of labels on each particle. SERS detection of free prostate-specific antigen (PSA) using this method showed a LOD of approximately 1 pg/mL in human serum and approximately 4 pg/mL in bovine serum. 

Silver staining can also be applied to increase sensitivity. Mirkin et al. [38] found that SERS signal of the labeled immunogold can be significantly enhanced by silver staining. Xu et al. [39] also reported a SERS immunoassay using probe-labeling immunogold nanoparticles with silver staining enhancement, the detection limit for Hepatitis B virus surface antigen was found to be as low as 0.5 mg/mL.

Another way to increase SERS sensitivity is the introduction of different shapes of nanoparticles. Shape dependent SERS intensity of Raman probes utilizing gold-Raman probe-silica sandwich as substrates has been investigated [40]. Li et al.’s result showed that among three types of gold cores (gold nanospheres, nanorods and nanostars), nanostars had the highest SERS enhancement factor while nanospheres had the lowest. However, issues such as batch-to-batch variations and long-term stability hamper the practical SERS application of noble metal nanoparticles with different shapes. 

Non-specific adsorption remains to be a big issue in immunoassay diagnosis. Systematic investigations by Porter et al. [41] showed that optimization of experimental parameters such as salt concentration, binding buffer, pH value and sample agitation can significantly reduce the nonspecific adsorption. A thin layer of SiO_2_ on noble nanoparticles can not only prevent the releasing of Raman tags, but can also played the similar role of albumin from bovine serum (BSA) in inhibiting the nonspecific bindings [42]. Kim et al. [43] showed that the agglomeration of AgNPs in highly concentrated buffer solutions could be prevented by the layer-by-layer deposition of cationic and anionic polyelectrolytes, and the nonspecific adsorption of proteins could be suppressed by the introduction of poly(ethylene glycol) into the immunoassay. Chuong et al. [44] linked one Raman reporter to immunogold and another to a gold film, the binding of target protein can create a “hot spot” with strong SERS spectra from both Raman reporter molecules, whereas signals generated by nonspecific binding lack one or the other label. By this way they can efficiently distinguish true from false positive results.

The introduction of magnetic nanoparticles can help to increase the sensitivity and decrease the nonspecific adsorption at the same time. Magnetic nanoparticles can not only facilitate highly efficient analyte separation and enrichment, but can also generate enough hot spots for SERS signal enhancement. What’s more, since all reactions occur in solution, this can also solve the problem of slow diffusion-limited immunoreaction on a traditional solid substrate [45]. SERS-based sandwich immunoassay with antibody coated magnetic nanoparticles have been applied in the detection of IgG (LOD 1–10 ng/mL) [46], lung cancer marker carcinoembryonic antigen (CEA, LOD 1–10 pg/mL) [45], *Escherichia coli* (LOD 8 cfu/mL, cfu represents colony-forming units) [47], etc. we also developed a magnetic immunosensor based on SERS spectroscopy to detect intact but inactivated influenza virus H3N2 (A/Shanghai/4084T/2012). This immunosensor could detect H3N2 down to 10^2^ TCID_50_/mL (TCID_50_ refers to tissue culture infection dose at 50% end point) [48]. 

## 4. Aptamer-SERS

Aptamers are mainly oligonucleotide molecules that can bind to specific target by folding into specific three-dimensional (3D) structures [49], which are mainly screened by Systematic Evolution of Ligands by Exponential Enrichment (SELEX). The strong aptamer−analyte interaction can enhance the specific recognition and enrichment of analyte from mixture samples. Compared with antibodies, aptamers have their special advantages in recognition, such as cost-effective synthesis, high binding affinities for specific targets [50], mild reaction condition, tolerance to internal labeling [51] and good stability [52]. What’s more, some small molecules do not have the corresponding antibodies due to the lack of immunoreaction. On the contrary, aptamers is more suitable for small molecules recognition by 3D DNA/RNA strand folding after aptamer-analyte interaction. Therefore, the range of analytes that can be detected by aptasensor is broader than that of antibody based technique. Furthermore, aptamers can be produced in a large quantity with high reproducibility and purity [53]. 

In SERS-based aptasensor, aptamers are introduced via electrostatic interaction [54] or covalent binding on Ag or Au of diverse sizes and morphologies (nanoparticles [55,56], nanostars [57], nanorods [58,59] or other irregular Ag/Au surface). Aptamers can bind to various targets, such as ions, small molecules, proteins, nucleic acids, virus, cells and even bacteria, tissues and organisms. When the analytes can provide distinct Raman signals of their own, they can be detected directly [60]. Otherwise, analyte information is indirectly acquired by the SERS signal changes of the Raman tag-labeled aptamers [61]. 

### 4.1. Metal Ion 

**Figure 5:**
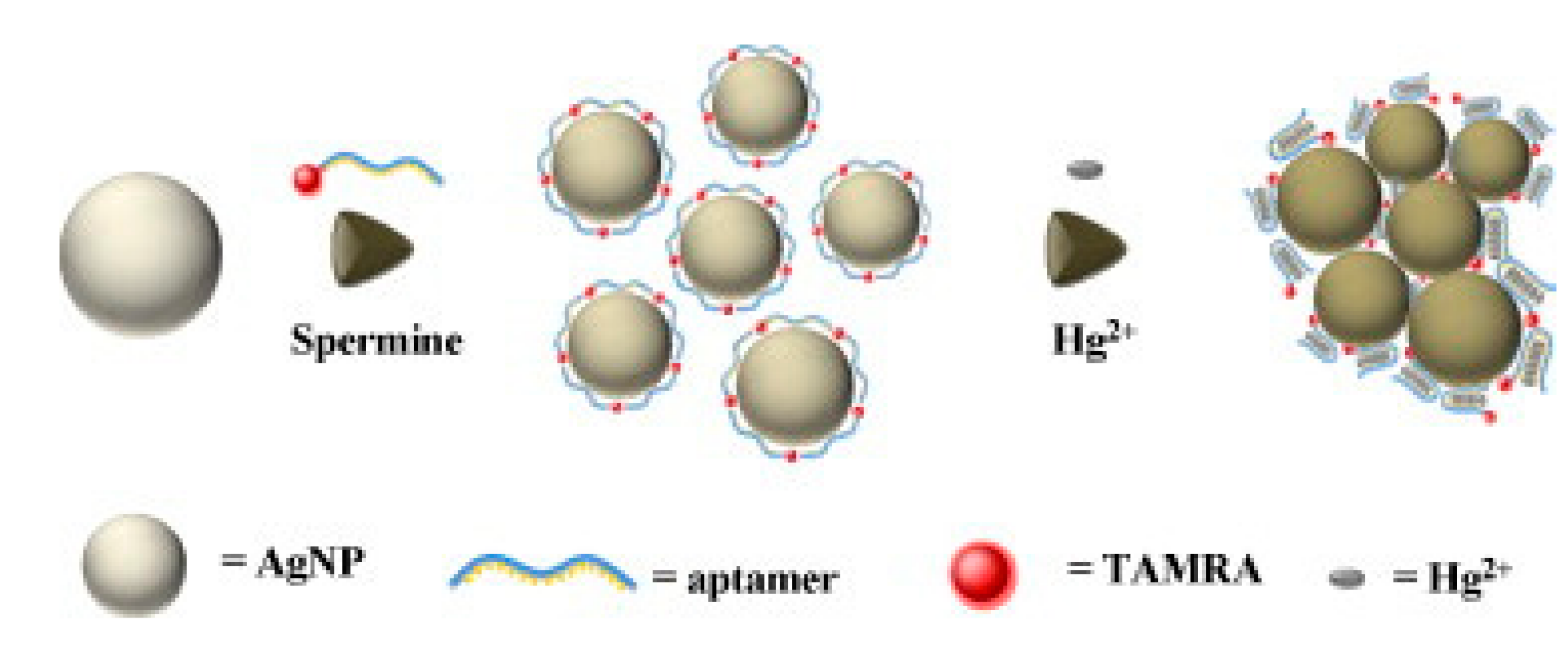
The formation of thymine (T)-Hg^2+^-T caused the aggregation of AgNPs [65].

**Figure 6:**
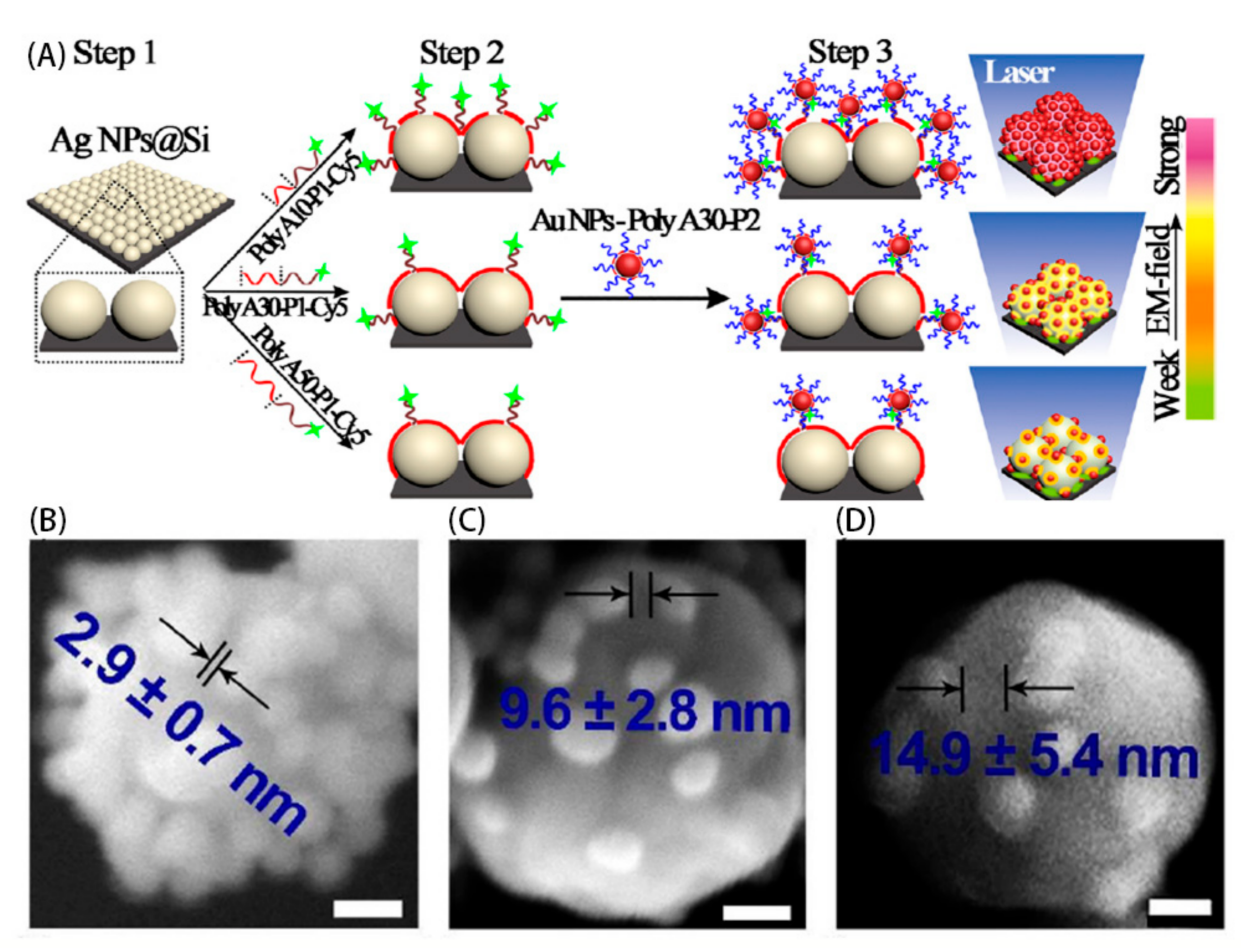
(**A**) A polyadenine mediated core-satellite nanoassembly immobilized on the silicon wafer [66]. (**B**–**D**) are SEM images of the interparticle gaps of satellite Au NPs of Au-Au NPs mediated by Poly A10, Poly A30, and Poly A50.

Heavy metal ion pollution is one of the most serious problems in recent years. SERS-based aptasensor has already been applied in the recognition of many metal ions such as Pb^2+^ [62], As^3+^ [63,64] and Hg^2+^. Wang et al. [65] used a SERS-based aptasensor to detect Hg^2+^ (Figure 5). The formation of thymine (T)-Hg^2+^-T caused the aggregation of AgNPs, which leads to the increased number of hot spots and enhanced Raman signals. This simple method shows high sensitivity (LOD is 5 nM) and selectivity. Zhu et al. [66] designed a polyadenine (Poly A)-mediated core-satellite nanoassembly to detect Hg^2+^ in a real system with good selectivity and sensitivity (LOD is 100 fM). The nanoassembly structure can be adjusted by altering the length of Poly A block (Figure 6). A bases in the Poly A tail were completely adsorbed on core Au NPs. Therefore, Poly A tails could provide anchors as well as density control for nonthiolated oligonucleotides. The reproducibility of this aptasensor can be further improved by immobilizing those nanoassemblies on a silicon wafer. Kang et al. [67] immobilized tetramethylrhodamine (TMR)-labeled aptamers onto the AgNPs. After anchoring the AgNPs on glycidyl methacrylate-ethylene dimethacrylate (GMA-EDMA) substrates by thiol group, this substrate showed high stability and reproducibility in Hg^2+^ detection with a LOD of 2.5 nM.

### 4.2. Small Molecule 

Pesticides are essential in modern agriculture practices [68]. Pang et al. [69] design a SERS-based aptasensor that can detect four pesticides simultaneous in apple juice. The aptamer-linked-Ag dendrites were modified with 6-mercaptohexanol to cut down nonspecific binding. Different pesticides can be detected based on their own fingerprint Raman spectra. The LOD of profenofos, phorate, omethoate and isocarbophos are 5 ppm (14 μM), 0.1 ppm (0.4 μM), 5 ppm (24 μM) and 1 ppm (3.4 μM), respectively.

Adenosine triphosphate (ATP) plays an important role in life system. Chen et al. [70] employed a Poly A mediated nanostructure to detect ATP. The number of aptamers on a single AuNPs can be adjusted by the length of Poly A block. The LOD is 10 μM. This system can also be used in intracellular ATP molecules Raman mapping. 

**Figure 7:**
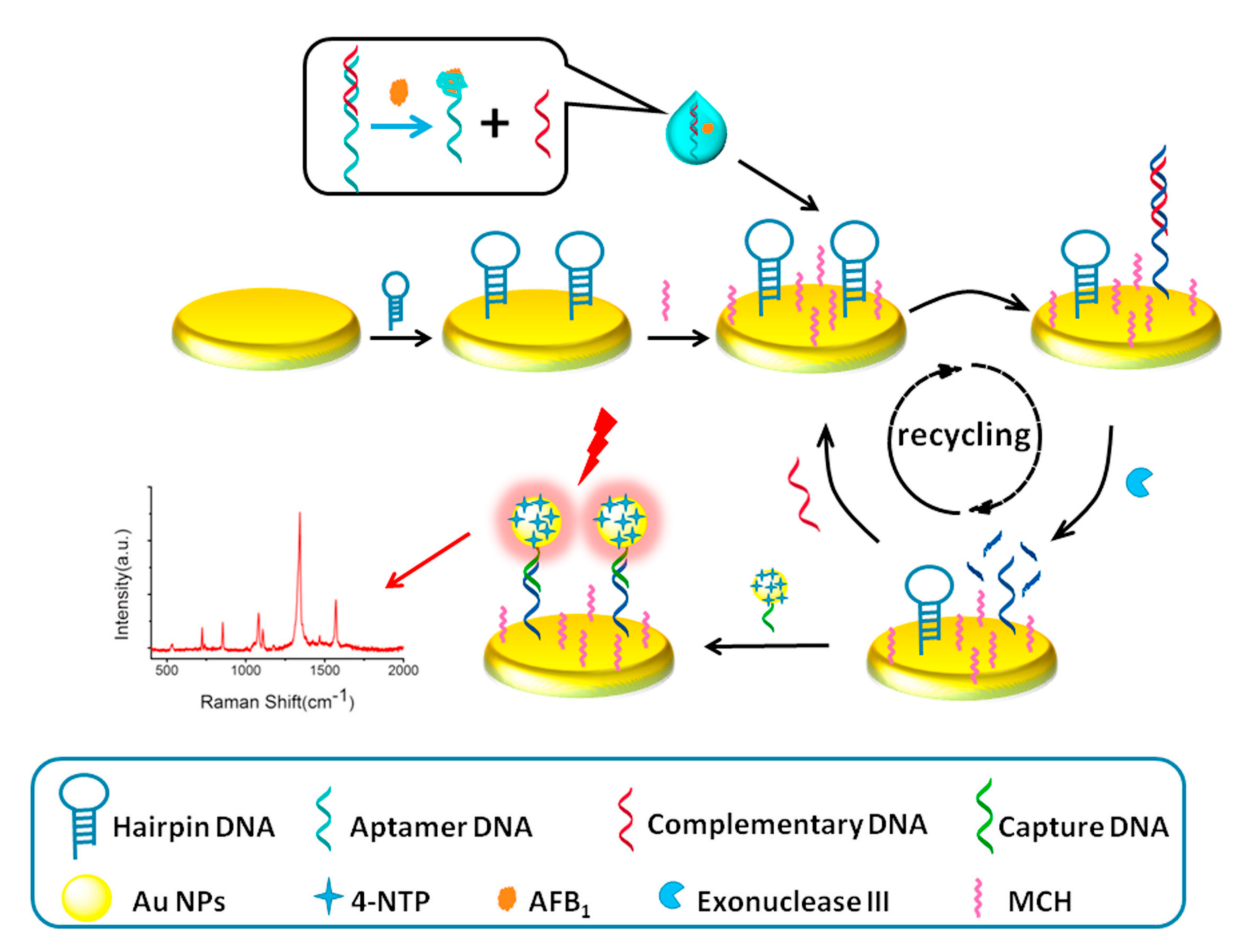
A recycling SERS-based aptasensor chip using the exonuclease to amplify the Raman signal [73].

Mycotoxins can make damage to the nervous, immune and reproductive system. Aflatoxin B1 (AFB1) is common found in foods and feeds stuffs, which is the most toxic one in the aflatoxin family [71]. Li et al. [72] fabricated a SERS-based aptasensor based on gold nanostar core-silver nanoparticles satellites to detect AFB1 in liquid. The Raman tag is modified on the surface of silver satellites. When the AFB1 is present, the satellite nanoparticles will be attached to the gold core nanostar. Thus, the SERS signal will be enhanced. Li et al. [73] described a recycling SERS-based aptasensor chip for detecting AFB1 (Figure 7). To ensure the specificity and sensitivity, they enhanced the Raman signal by using the exonuclease. The LOD is calculated to be 0.4 fg/mL.

### 4.3. Biomacromolecules

As indicators/biomarkers of many diseases, the detection of biomacromolecules is essential in diagnosis and treatment. Prostate-specific antigen (PSA) is one of the several generally accepted organ-specific biomarkers. Yang et al. [74] designed a sandwich-like structure aptasensor to detection PSA selectively, the LOD can be as low as 5.0 pg/mL. However, the PSA is only organ-specific and no tumor-specific. To solve this practical problem, Hao et al. [75] designed a nanoparticle assembled pyramids structure, which can detect thrombin and PSA in the meantime. The LOD of PSA and thrombin is 3.2 × 10^−20^ M and 5.7 × 10^−17^ M, respectively. Although the system detects two different analysts at the same time, it still keeps selective and sensitive and has been applied in real sample diagnosis. 

**Figure 8:**
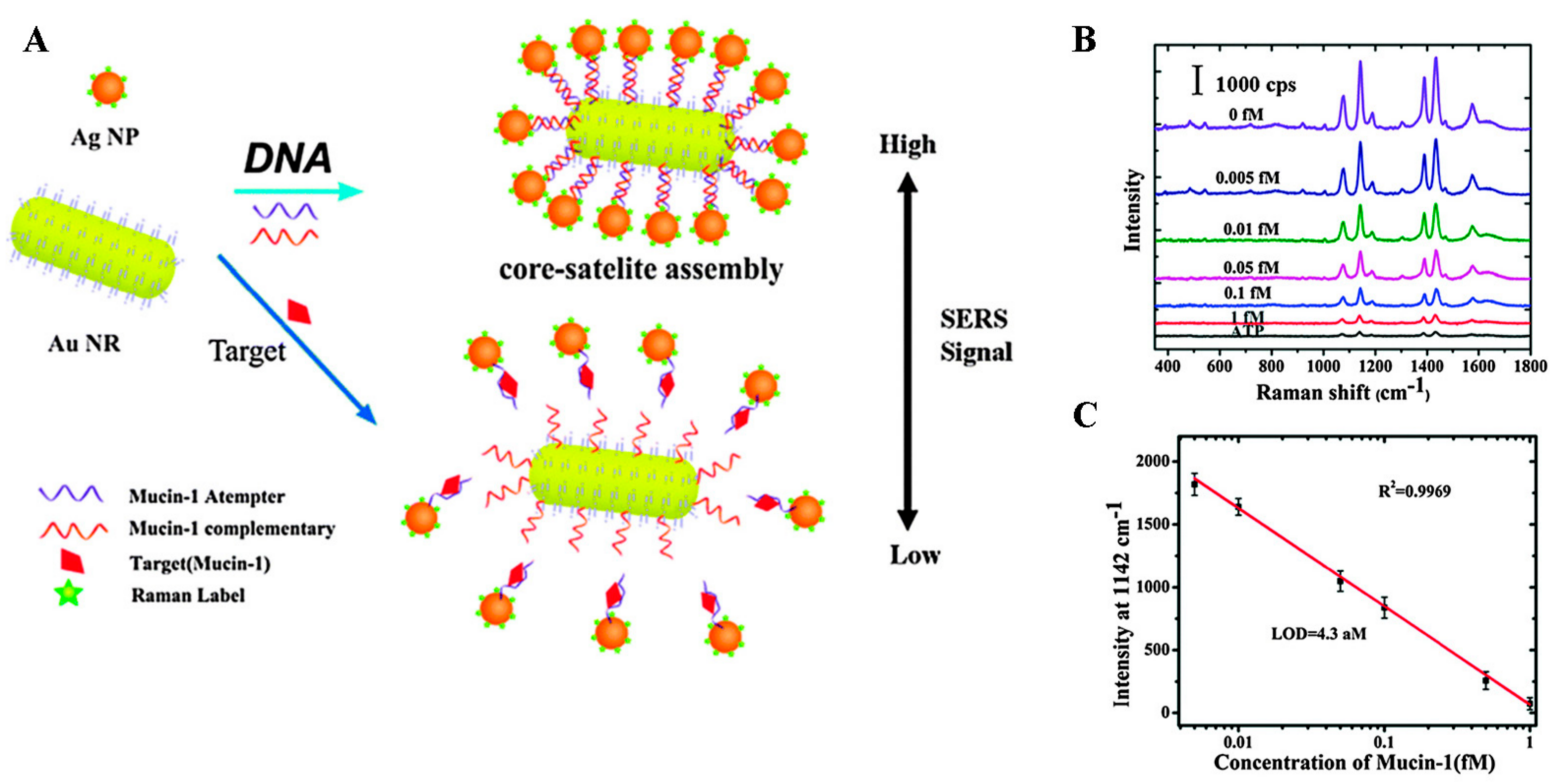
(**A**) Mucin-1 detection based on SERS with bimetallic core (gold nanorods)—satellite (silver nanoparticles) assemblies [77]. (**B**) SERS spectra of Mucin-1 detection; (**C**) Standard curve for Mucin-1 detection with corresponding peak intensities at 1142 cm^−1^.

Alpha fetoprotein (AFP) is a biomarker for germ cell tumor, hepatocellular carcinoma and certain gastric carcinomas. Wu et al. [76] use AgNPs-aptamer and its complementary strands to fabricate Ag-trimers for the detection of AFP. The existence of AFP caused the separation of the trimers, causing the decreased number of hot spots and SERS signal. The LOD of this signal-off system is 0.097 aM. Feng et al. [77] established a bimetallic core (gold nanorods)—satellite (silver nanoparticles) assemblies for the detection of a specific breast cancer marker protein, Mucin-1 (Figure 8). The existence of Mucin-1 will cause the separation of aptamer and its complementary strand, leading to the decreased number of hot spots and SERS intensity. The LOD is 4.3 aM. 

Different miRNA or DNA targets can also be detected simultaneously. Su et al. [78] synthesized mushroom-like Au-Ag composite nanoparticles with different aptamers. The formation of gap structures was mediated by the DNA and Raman tags inside the gap can be significantly enhanced. Multidetection can be achieved by anchoring different tags on different aptamers.

Aptamers have also been employed in SERS mapping as well. As is known to all that fingerprint is useful and sufficient for individual identification. In Zhao’s study [52] an aptamer-modified SiO_2_ shell is used to avoid interference of the external environment and identifying lysozyme, one of the polypeptide components universal in fingerprints.

### 4.4. Living Organisms

Pathogenic bacteria are an ineluctable point when it comes to security issues. Zhang et al. [79] used Fe_3_O_4_ magnetic NPs coated with two kinds of aptamers (for *S. typhimurium* and *S. aureus*). They also used two kinds of AuNPs, each was coated with one kind of aptamer (for either *S. typhimurium* or *S. aureus*) and Raman tag. The magnetic NPs will be linked with the AuNPs when the targets existed. The formation of a sandwich-like nanostructure caused the increased number of hot spots. The LOD is 15 cfu/mL and 35 cfu/mL for *S. typhimurium* and *S. aureus*, respectively.

Medical research and patient treatment rely on live optical imaging in many ways. Raman imaging overcomes some of the limitations of other methods [80]. Firstly, narrow peaks help identify various analytes simultaneously. Secondly, it can be excited by near infrared laser, with the advantage of imaging in deep tissue. What’s more, the superior signal stability is also beneficial. Huang et al. [58] demonstrated the use of aptamer-gold nanorods for targeted photothermal therapy. Deng et al. [81] traced the therapeutic process of targeted aptamer/drug conjugate on cancer cells. By real-time SERS spectra, the process is clearly recorded.

In addition to some organ-specific biomarkers, circulating tumor cells (CTCs) also have attracted more attention in medical biology, practice diagnosis and cancer treatment assessment. CTCs are cells that drop from a primary tumor and take part in body fluid circulation. Mocellin et al. [82] have proved that CTC status is correlated with both tumor-node-metastasis stage and survival. Wu et al. [83] presented a selective SERS method to CTC detection via using reductive bovine serum albumin and folic acid receptor. To provide the accuracy of detection, various aptamers are introduced into the lab test detection. Sun et al. [84] developed a rapid method to capture and identify of CTCs with specific aptamers. With the presence of magnetic beads and 4-mercaptobenzoic acid (4-MBA), SERS image can be obtained and the CTCs will be separated and enriched. The capture efficiency is 73% and 55% in buffer and whole blood sample, respectively. Compared with the traditional methods, a SERS-based aptasensor can trace the origin of the specific tumor and tumor metastasis. 

Representative studies of aptamer-SERS methods are summarized in Table 2, including SERS Mapping.

## 5. MIPs-SERS

The concept of molecularly imprinted polymers (MIPs) was proposed in 1972 by Wulff and Sarhan [86]. Molecular recognition site can be created by imprinting template molecules in a polymer matrix through the noncovalent/covalent interactions between templates and functional monomers. The removal of templates after polymerization generates the recognition sites complementary to the shape, size, and functionality of templates [87]. These recognition sites can selectivity rebind with template with high affinity, similar to those of antibody-antigen systems. Therefore, molecularly imprinted material has been called “artificial” antibody. It has the advantages of cavity predetermination and specific recognition. In addition, it shows much better stability than natural antibody [88].

**Figure 9:**
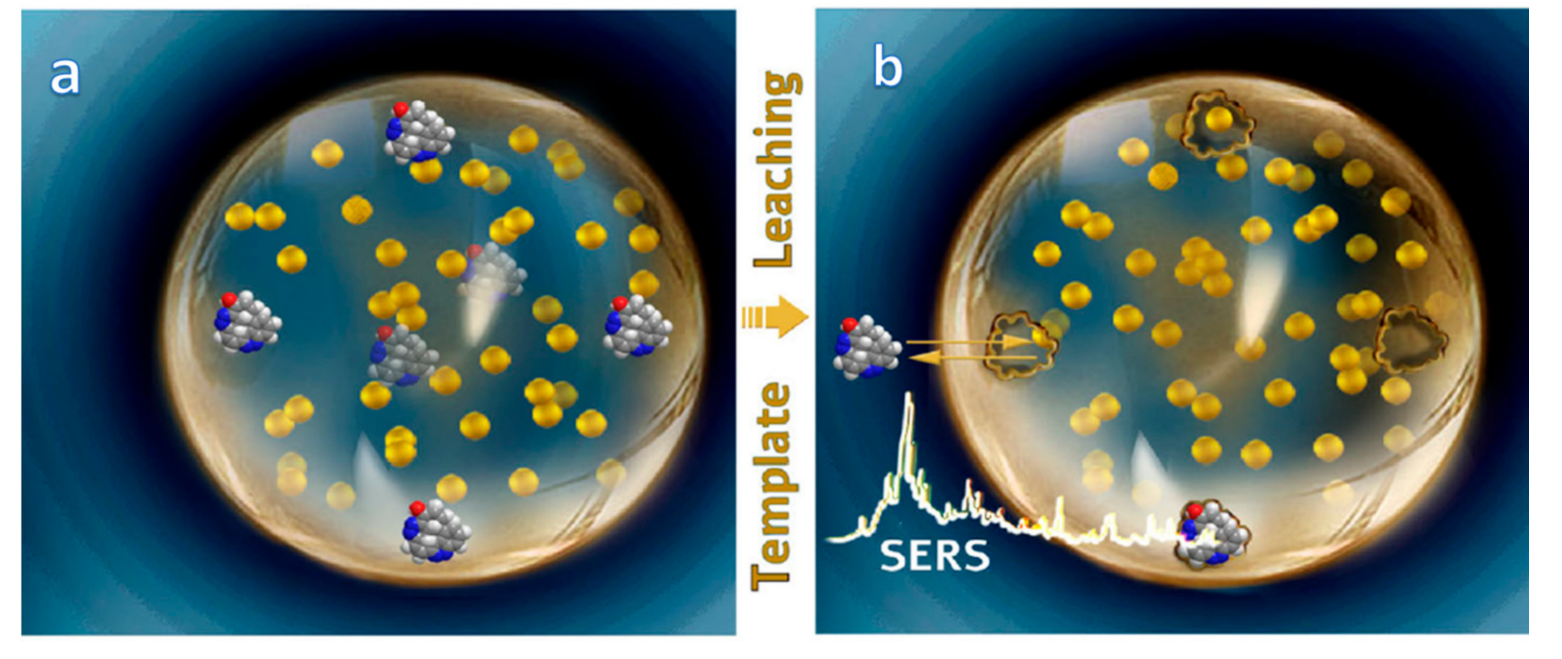
Schematic of the one-pot controlled synthesis of AuNPs@MIPs for improved SERS sensing (**a**) [89]. After the template removal, target molecules can selectively rebind with the cavities, and the analytes can closely contact with the Au NPs in the imprinted layer (**b**), which significantly improves the selectivity and sensitivity.

Combination of SERS with molecularly imprinted polymers (MIPs) is a promising analytical method to improve the selectivity of SERS technique. As shown in Figure 9, AuNPs as SERS substrate were doped in the MIPs and can be applied in the sensitive and specific SERS detection [89]. Combining the specific selectivity of MIPs with the high-sensitivity offered by SERS has been proven to be a very powerful tool for the detection of specific analyte in complex surroundings.

The simplest MIPs-SERS system is the analyte extraction/enrichment by MIPs and then rinsed out for SERS detection. In this strategy, MIPs only play a role as solid phase extraction (SPE) reagents with molecular recognition cavities for the specific enrichment of organic compounds from a complex matrix [90]. For example, MIPs-SERS/colorimetric dual sensor was developed to determinate chlorpyrifos (CPF) in apple juice [91]. MIPs particles were synthesized by bulk polymerization and packed into SPE column to selectively adsorb and separate CPF from apple juice. The enriched CPF in the column was then eluted and mixed with AgNPs for SERS/colorimetric detection. In this process AgNPs acted as both color indicator and SERS-active substrates. This method can detect CPF in apple juice with concentration as low as 0.01 mg/L. Similarly, other groups used MIP-based SPE/SERS to detect trace levels of six Sudan dyes in chili powder samples [92], melamine adulteration in milk [93], chloramphenicol residues in honey and milk [94] and α-tocopherol in vegetable oils [95].

MIP-based SPE can effectively remove impurities and interfering substances [96]. The enriched analyte were then extracted out and mixed with SERS substrates (such as AuNPs or AgNPs) for SERS detection. However, some analytes might have poor affinity with SERS substrates, and it is also difficult for hydrophobic analytes to adsorbed evenly on hydrophilic SERS substrates, both will affect the final SERS result [93]. An integrated MIPs system in which the SERS substrates are embedded in the MIPs can address such issues. This can be done by bringing plasmonic nanoparticles or introducing their precursors into the MIPs polymerization process followed by precursor reduction and template removal. This “one step” MIPs-SERS sensor can enrich and detect target analyte at the same time, showing better analysis efficiency. Liu [97] developed SERS-based sensor for the determination of theophylline by imprinting the target molecules on the surface of in-situ reduced AgNPs. The desired recognition sites after template removal were homogeneously distributed on the AgNPs. Similarly, Hu [93] used this one-step approach MIPs-SERS approach to rapidly detect melamine in tap water and milk.

**Figure 10:**
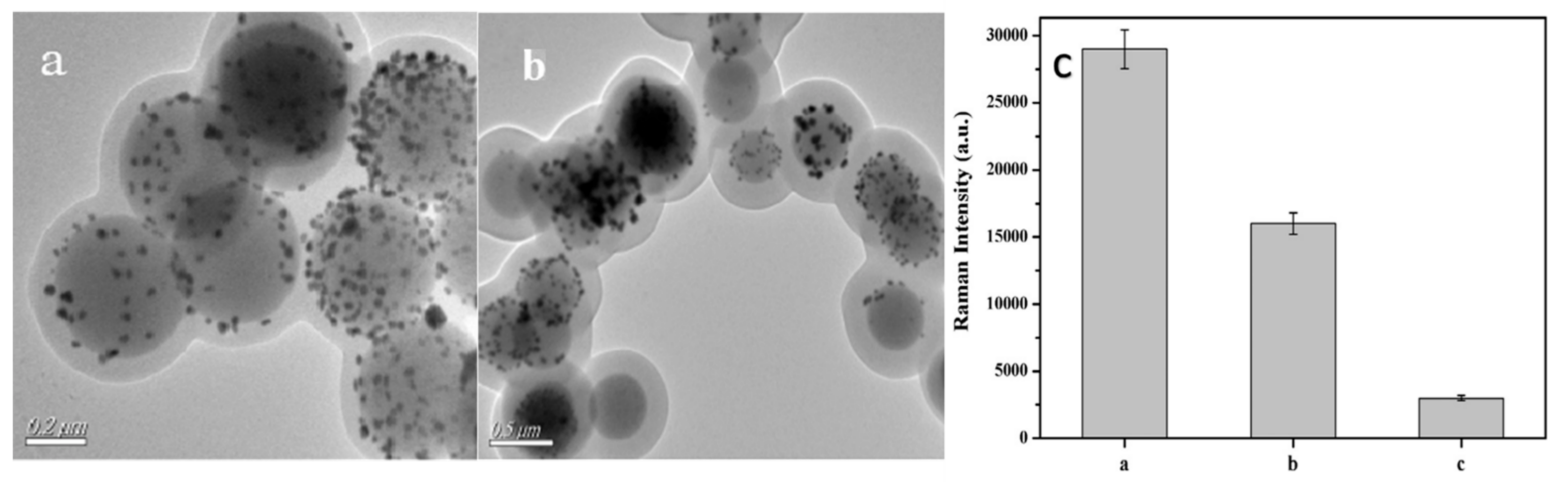
The TEM images of SiO_2_/Ag/MIPs with different shell thickness: 40 nm (**a**), 170 nm (**b**) and (**c**) the SERS detections of different thicknesses of SiO2/Ag/MIPs to the same concentration of R6G at 10^−6^ mol L^−1^ (40 nm (**a**), 100 nm (**b**), 170 nm (**c**)) [106].

MIPs fabricated by bulk polymerization need to be pulverized, ground and sieved. To overcome the disadvantage of time-consuming step, alternative forms of MIPs such as MIP monolithic columns [98,99,100] (prepared by in situ polymerization directly inside columns) and thin films [101,102,103] become increasingly attractive for efficient affinity separations. Jia [104] developed a molecularly imprinted composite membrane for SERS rapid detection of hypoglycemic agents. The molecularly imprinted membrane (MIM) was prepared by using filter paper as a carrier, and Ag NPs were added before the polymerization to simplify the process. Although the LOD is only 5 mg/L, it is a good attempt to simplify MIPs-SERS fabrication. Some researchers proposed the concept of surface molecularly imprinting(SMI), in which most of the binding sites are distributed on the particle surface [105]. The most well-known among them is the MIP core-shell structure. For example, a highly-controllable core-shell SERS sensor was developed to detection of R6G in water [106]. AgNPs as the SERS substrates were in-situ reduced on amino-functionalized SiO_2_ followed by polymerization to for MIPs shell. The shell thickness can be tuned by the concentration of EGDMA. When the shell thickness was 40 nm the SERS signal was the strongest. With the increase of shell thickness the SERS signal weakened, proving that Raman signal of analytes adjacent to rather than far away from SERS substrates can be significantly enhanced (Figure 10). The LOD of SiO_2_/Ag/MIPs for R6G was 10^−12^ mol/L. The Raman intensity of R6G on SiO_2_/Ag/MIPs was much stronger than that of RB and CV under the same concentration. Similarly, Xue [107] fabricated surface-imprinted core-shell gold nanoparticles for selective detection of bisphenol A by using the core–shell technique. Gold nanoparticle was the core and then a MIP layer was anchored on the surface of Au NPs directly to form MIP-Au NPs. 

Core-shell structured MIPs-SERS platform has many merits. In those irregular MIPs-SERS systems the complete extraction of templates which were deep inside the bulk materials is quite difficult due to the high cross-linking nature of MIPs [108,109,110]. Some templates have strong SERS signal. Therefore, the remained template might strongly influence the final SERS result. Some strategies such as pseudo template, synthetic structural analogues and dummy molecularly imprinted polymers (DMIPs) [111,112] was introduced to address this issue in bulk materials. On the contrary, templates within the thin shells of core-shell MIPs-SERS sensors can be completely removed to form effective recognition sites. What’s more, core-shell nanoparticles possess good dispersibility, high surface-to-volume ratio and rapid binding kinetics [113,114].

**Figure 11:**
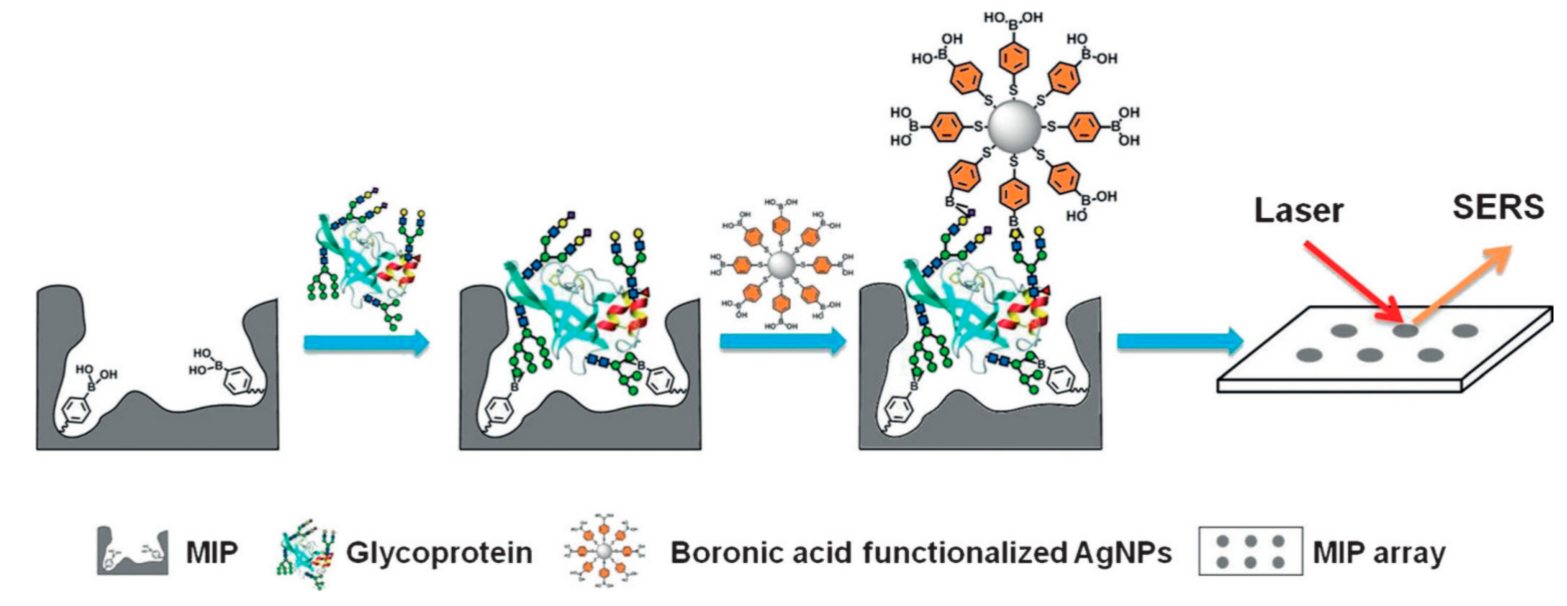
Schematic representation of the boronate-affinity sandwich assay of glycoproteins [122]. Target glycoprotein can be captured specifically by a boronate-affinity MIP array. The captured glycoprotein is then labeled with boronate functionalized AgNPs for SERS detection.

So far most imprinting targets are small molecules, such as pesticide [115,116], additives [92,93], and metal ions [117,118]. However, detection of bacteria or large proteins such as toxins and hormones [119] in organisms has severe challenges [120]. Imprinting of proteins represents a major challenge that arises from their large size and multiplicity of functionalities [121] and poor stability during polymerizaton. Ye et al. [122] reported a boronate-affinity sandwich assay (BASA) for the specific and sensitive detection of trace glycoproteins. BASA relies on the formation of sandwiches between boronate-affinity molecularly imprinted polymers, target glycoproteins, and boronate-affinity SERS probes (Figure 11). The feasibility of this method in real application was proved by the determination of trace alpha fetoprotein (AFP) in human serum. The whole process only takes 30 min. Lv et al. prepared MIPs-SERS sensors by the self-polymerization of dopamine on the surface of gold nanorods to detect protein biomarkers .The feasibility of this new approach is demonstrated with detection of transferrin in human serum [123]. Representative studies of MIPs-SERS methods are summarized in Table 3.

## 6. Microfluidics-SERS

Microfluidic device is a platform that can achieve the miniaturization, integration and automation of analysis processes. Operations such as centrifugation, mixing, separation, reaction and detection can all be integrated in a microfluidic device. Microfluidic systems can couple with different detectors for accurate and reliable detection, characterization and diagnosis. SERS is a newly emerged detection technique in microfluidic devices due to its advantages such as non-destructiveness, highly sensitivity, rapidity and low aqueous interference. Combination of SERS with microfluidic techniques (Microfluidics-SERS) can manipulate and detect small amount of samples in a rapid, sensitive, reproducible and high-throughput mode.

**Figure 12:**
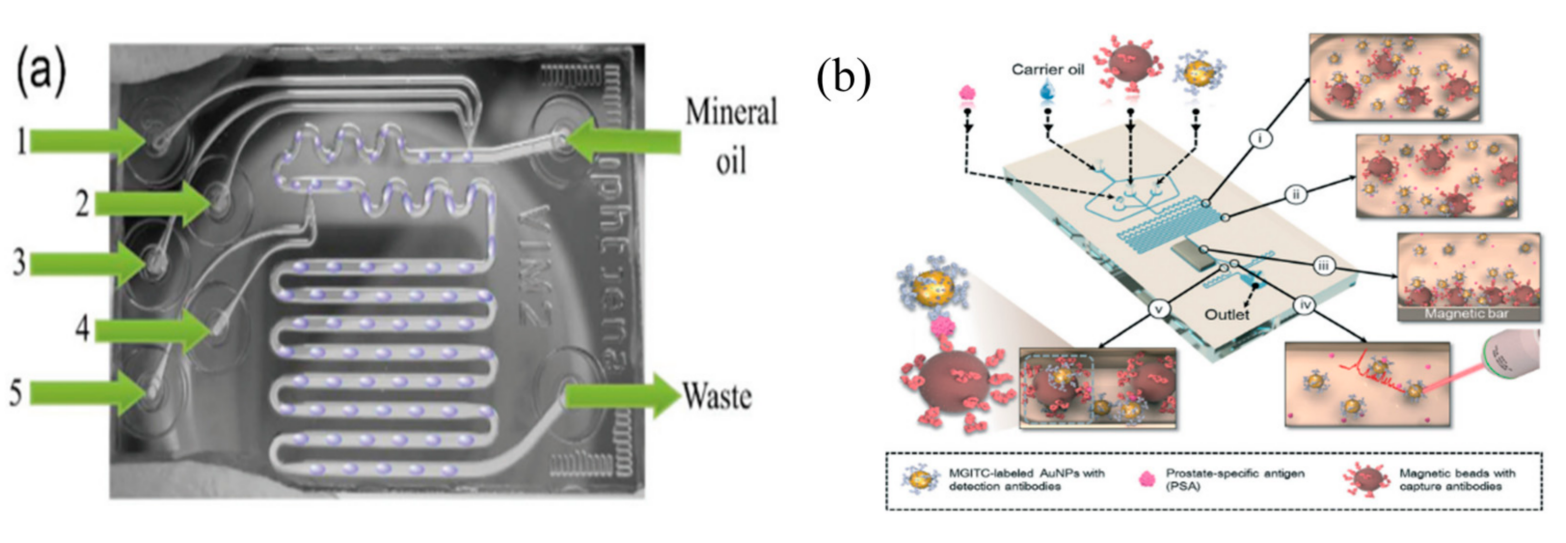
A typical microdroplet base SERS sensing platform: ports 1–5 are used for the injection of an aqueous solution containing the sample, the SERS active substrates, and their aggregation agent [126]; (**b**) Schematic illustration of the SERS-based microdroplet sensor for wash-free magnetic immunoassay. The sensor is composed of five compartments with the following functions: (i) droplet generation and reagent mixing; (ii) formation of magnetic immunocomplexes; (iii) magnetic barmediated isolation of immunocomplexes; (iv) generation of larger droplets containing the supernatant for SERS detection and (v) generation of smaller droplets containing magnetic immunocomplexes [129].

Microfluidic platforms can be divided into five groups according to different liquid propulsion forces: pressure, capillary, centrifugal, electrokinetic and acoustic [124]. Among them pressure, capillary and centrifugal driven microfluidics are the most familiar ones. Pressure-driven microfluidic devices mainly include laminar flow, segmented flow and continuous flow microfluidic devices, in which the segmented flow microfluidics have already been applied in conjunction with SERS. Droplet-based microfluidic chips are the most popular in segmented flow microfluidic device [125]. A typical droplet-based microfluidic-SERS platform is depicted in Figure 12a. Aqueous droplets are generated in the continuous phase of the mineral oil. AgNPs and KCl solution or H_2_O are injected in the existing droplets. The mixing of the analytes is assured by two meandering channels. The SERS spectra were acquired continuously with fixed integration time in the channel [126]. Popp’s [127] group has done a lot of works on the application of SERS detection technique in a liquid/liquid segmented microfluidic system. Their results proved that combination of segmented microfluidic system with highly sensitive SERS technique results in a reproducible quantification of analytes. They found that the introduction of analytes’ deuterated isotopomers as internal standards in the liquid–liquid two phase segmented flow lead to reproducible and comparable SERS results independent from the used colloid [128]. A novel wash-free magnetic immunoassay technique for prostate-specific antigen (PSA) detection that uses a SERS-based microdroplet sensor is depicted in Figure 12b, the separation process through a magnetic bar is embedded in the droplet-based microfluidic system. This approach is expected to be useful as a potential clinical tool for the early diagnosis of prostate cancer [129]. 

**Figure 13:**
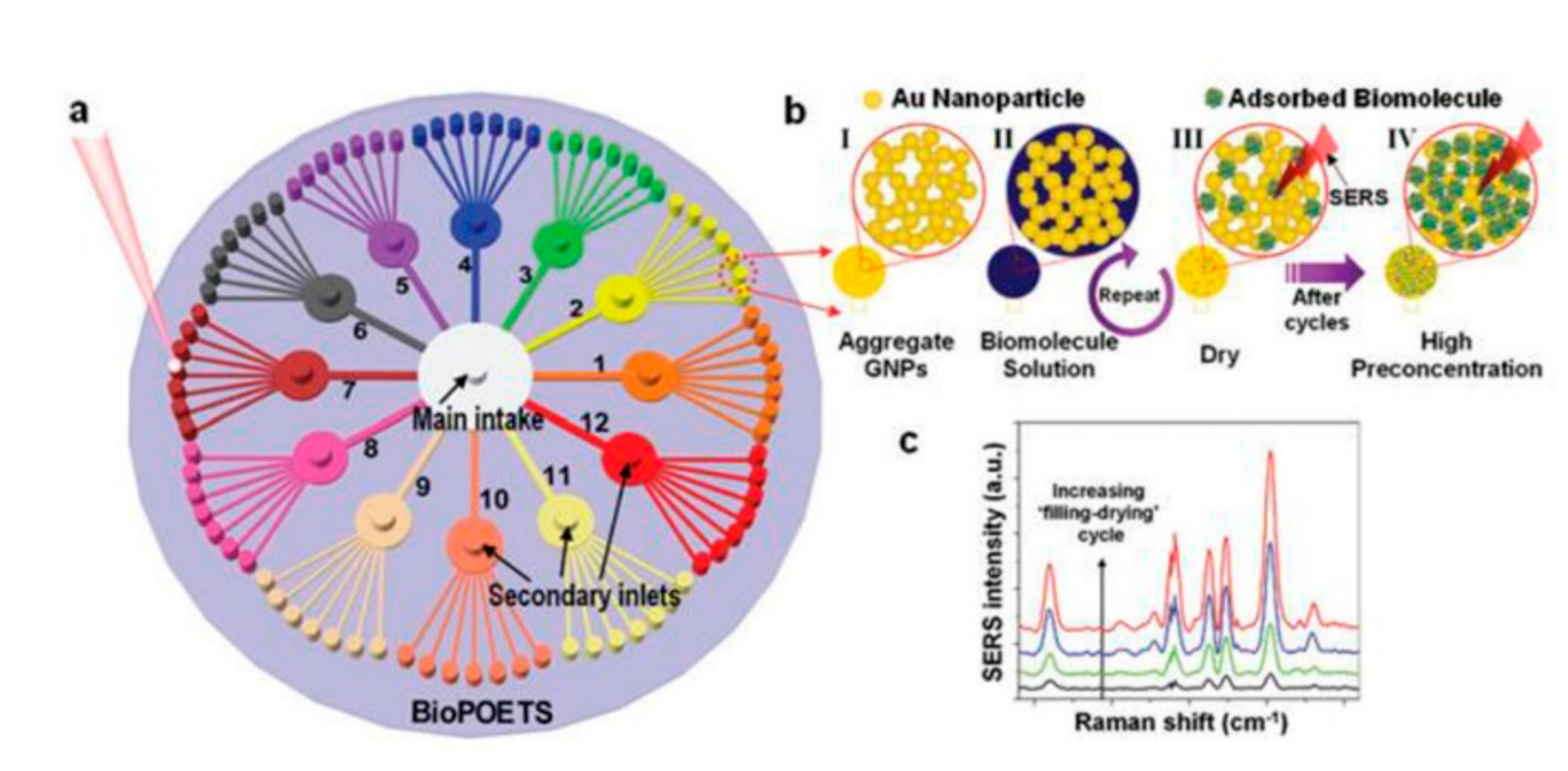
Optofluidic SERS-CD platform and preconcentration mechanism. (**a**) Schematic illustration of the optofluidic SERS-CD platform with high-throughput sample preparation; (**b**) The mechanism of preconcentration consisting of 4 steps. I: Fabrication of SERS-active sites; II: Injection of a sample solution; III: Adsorption of target molecules on the SERS-active site during drying process prior to SERS measurement; IV: Accumulation of the adsorbed molecules by the repetition of the steps II and III. (**c**) The enhanced SERS intensity due to the preconcentration of the target molecule with increasing ‘filling–drying’ cycles [131].

Centrifugal microfluidics is a rapidly growing lab-on-a-chip technology, in which fluid movement is achieved by spinning a disc [130]. A series of processes include metering, switching, aliquoting, etc. [124] are controlled by the frequency protocol of a rotating microstructured substrate within centrifugal microfluidics. The first paper which combined centrifugal microfluidics with SERS technique took advantages of the centrifugal force of a rotational CD to effectively concentrate analytes for effective label-free environmental and biomolecular SERS detections. The CD-based SERS platform is shown in Figure 13. This kind of CD-based SERS platform provide high-throughput, high-sensitive and large-area uniform SERS substrates for ultrasensitive label-free sensing [131]. Another report showed that ionic wind near a corona needle tip above a small liquid reservoir can generate microcentrifugal flows and trap suspended bacteria within a few minutes. Bacteria decorated with antibody-functionalized SERS micro-tags were concentrated and enriched at the reservoir bottom and can then be recognized and quantified by SERS signals from the tags [132]. 

Conventional microfluidic fabrication included photolithography, electron beam lithography, printing, and molding. These steps usually involved high-cost technology and skilled operation with expert technicians. However, low-cost microfluidic devices are welcomed in real applications. Therefore many researchers have focused on the fabrication of paper-based microfluidics [125]. The liquids in paper-based microfluidics, like in other capillary microfluidic system, are driven by capillary forces [124]. Paper microfluidic devices can be designed into various types depending on their applications. Conventional fabrication methods of 2D paper-based microfluidic devices include wax printing, digital inkjet printing, photolithography, flexographic printing, plasma treatment, laser treatment, wet etching and wax screen-printing. Conventional materials for 2D paper-based microfluidic devices include cellulose-based filter papers and membranes. White’s group used filter paper as the substrate, AgNPs were printed onto the substrate by an inexpensive inkjet printer to form sensing arrays [133]. In this way they fabricated a very simple paper SERS devices that enable the concentration of analyte molecules into a small detection volume [134]. The measurements showed that the technique is quantitative. In addition to the paper substrate, nitrocellulose membrane has high trapping ability for protein and other biological macromolecules. Therefore, without any pretreatment procedure, SERS detection of glucose in blood samples can be directly carried out on the hydrophilic channel of the nitrocellulose membrane [135].

**Figure 14:**
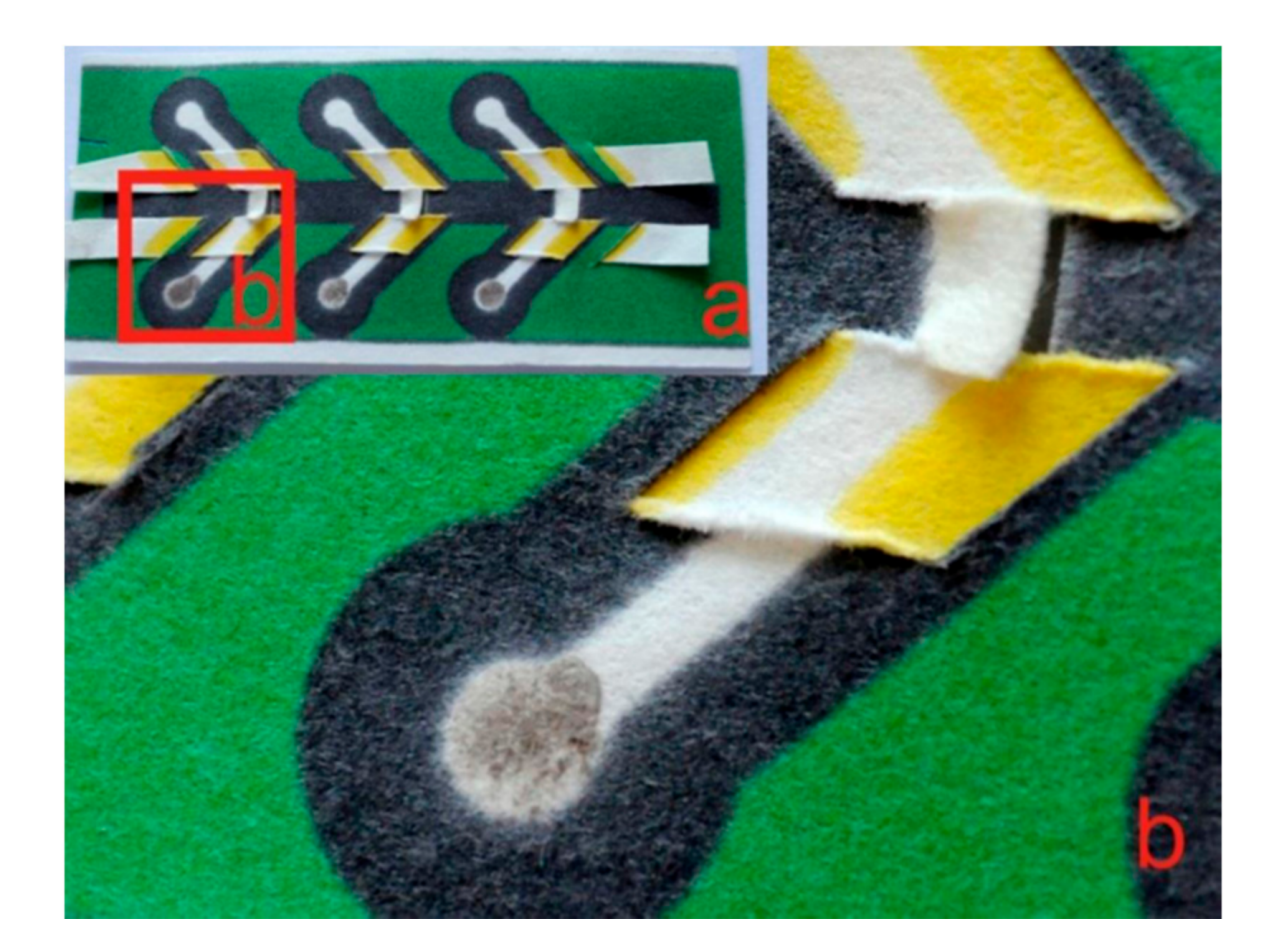
(**a**) The photograph of the switch-on-chip and (**b**) the enlarged view of the inlet and the pretreatment zone [139].

Conventional fabrication method of 3D paper-based microfluidic devices is stacking alternating layers of paper and water-impermeable double-sided adhesive tape. Details of each fabrication technique of microfluidic paper-based analytical devices can be found in a recent review [136]. 3D paper-based microfluidic devices were firstly proposed by Whitesides’ group [137]. Another studiers [138] also report fabrication of 3D paper-based microfluidic devices based on the principles of origami. The entire photolithographic process is completed by a hot plate, UV lamp, and a mask produced on a printer. A switch-on-chip multilayered microfluidic paper-based analysis device was fabricated by Luo et al. as a portable SERS immunoassay to detect clenbuterol from swine hair. Antigen was directly anchored in pattern on the paper chip, as shown in Figure 14. Unwieldy multistep operations in traditional immunoassay were simplified, which make this kind of paper chip very attractive for fast, cheap, accurate and on-site specific detection of clenbuterol from real samples [139]. 

Microfluidic-SERS technique has the advantages of low sample doses needed, multiplexing of microchannels, rapid and sensitive analysis, on-chip detection, etc. The incorporation of selective/specific recognition reagents (such as antibody, aptamer or MIPs) into microfluidic-SERS will make microfluidic-SERS chip a very promising platform for fast, ultrasensitive and specific analyte detection. Up to now the incorporation of antibody into the microfluidic-SERS system has been reported by many groups. For example, Zou et al. [140] reported the CEA detection in raw blood samples exploiting microfluidics. After capturing CEA by antibody coated magnetic nanoparticle, the immunocomplex were then mixed with AuNPs coated with both antibody and Raman tag to detect CEA in whole blood with picomolar precision. Cui et al. [141] studied cell–cell communications in situ with surface enhanced Raman scattering (SERS) nanoprobes and microfluidic networks for screening of immunotherapeutic drugs. Multiple immunosuppressive proteins secreted by cancer cells in a tumor microenvironment were quantitatively analyzed by a SERS-assisted 3D barcode immunoassay with high sensitivity (1 ng/mL). 

There were also some reports on the incorporation of aptamers into microfluidic-SERS systems. Catala et al. [142] reported the on-chip SERS quantification of *Staphylococcus aureus* in human fluids. SERS-encoded particles were functionalized with either an antibody or an aptamer can form a dense collection of electromagnetic hot spots on the surface of *S. aureus*. Quantification is achieved by passing the bacteria-nanoparticle aggregates through a microfluidic device with a collection/ detection window. They found that aptamers show much larger affinity for bacteria than antibodies. Therefore the final SERS intensity for aptamers is larger than that for antibodies. Fu et al. [143] reported the sensitive and selective polychlorinated biphenyls detection on a aptamer-based microfluidic-SERS sensor. Mercapto aptamer of 3,3′,4,4′-tetrachlorobiphenyl (PCB77) can capture PCB77 and immobilized on Ag nanocrown array detection zone to improve both sensitivity and selectivity. Chung et al. [144] detected trace mercury(II) ions using aptamer-modified Au/Ag core-shell nanoparticles by SERS technique in a microdroplet chip. The LOD was below 10 pM.

## 7. Final Statements

SERS itself is a fast, ultrasensitive detection technique, however, in complicated matrix the “fingerprint” recognition capability of SERS may fail due to the multiple interferences. Therefore, selective/specific detection is crucial for the real application of SERS technique, this can be done by the 5 approaches mentioned above. Chemical reaction-SERS method is more suitable for those analytes possessing little affinity to SERS substrate or having small Raman cross sections. However, the limited choice of reaction types hampered its wide application. It is hard to tell which of the three specific recognition approaches (antibody, aptamer and MIPs) is more effective. Antibodies have been widely used in immunoassays due to their strong recognition and binding capability. However, the modification reaction and the unfavorable condition may deteriorate their recognition capability. Compared to antibodies, aptamers show better batch-to-batch consistency and are more robust in different environments. As to MIPs, though the theoretical recognition and binding capability is not as good as antibody or aptamer, it’s the only technique among the 3 that can be applied in extreme conditions or non-aqueous environments. 

Microfluidics-SERS may hold the greatest promise among all the approaches mentioned in this review. The reason is manifold. Firstly, by careful chip design and fine parameter control, the best recognition/detection result and repeatability can be guaranteed. Secondly, by simple modification (surface decoration, stationary phase incorporation) the microchannels of the microfluidic device can effectively separate different analytes according to chromatography principles (size, charge, hydrophobicity and affinity) [145]. SERS detection can then be performed on places where analytes locate to achieve selective/specific detection. However, there’s only a few report on on-chip separation and SERS detection [146]. Thirdly, it’s easy for microfluidic chips to incorporate with other specific recognition technique, which has already been mentioned in Section 6. 

In conclusion, SERS is a rapid, sensitive and non-destructive detection tool. With the development of Raman spectroscopy fabrication technique, more powerful portable Raman machine has been introduced with cheaper prices. This makes SERS more favorable in in-field detection. Selective/specific recognition techniques such as chemical reaction, antibody, aptamer, MIPs and microfluidic devices can address the issues of multiple interferences during SERS detection. Together with the selective/specific recognition techniques, SERS may find broader application in real application, especially in high-throughput screening, quality control, in-field environmental monitoring and point-of-care testing.

## Figures and Tables

**Table 1:** Representative studies of reaction-SERS methods.

Analytes	Reagents	Substrates	Matrix	LOD ^1^	Linear Range ^2^	Ref.
hydrazine	*ortho*-phthaldialdehyde	AgNPs	water	8.5 × 10^−11^ M	10^−5^–10^−10^ M	[6]
ethylene and SO_2_	bromine-thiourea and OPA-NH_4_^+^, respectively	AuNPs	fruits	1.7 μg/L and 12.0 μg/L, respectively	14.7–21.1 μg/L and 35.1–35.9 μg/L	[7]
formaldehyde	4-amino-5-hydrazino-3-mercapto-1,2,4-triazole	AgNPs	water and food	0.15 μg/L	1–1000 μg/L	[18]
formaldehyde	3-Methyl-2-benzothiazolinone hydrazone	Au/SiO_2_ NPs	aquatic products	0.17μg/L	0.40–4.8 μg/L	[20]
acetone	2,4-dinitrophenylhydrazine	iodide modified Ag nanoparticles (Ag IMNPs)	urine	0.09 mM	9.6–96 mg/L	[17]
malondialdehyde	thiobarbituric acid	AgNPs		0.45 nM	0.45–4.5 μM	[21]
sodium dithionite	bis[4,4′-dithiodiphenyl azo-phenol]	A single cabbage-like Au microparticle	industry	0.08 μM	0.5–20 μM	[22]
acetone	4-(methylthio)benzaldehyde	Ag@AuNCs				[24]
HNO_3_ and H_2_SO_4_	ammonium hydroxide	Klarite substrate		100 ppb		[25]
NO	*o*-phenylenediamine	AuNPs	living cells	10^−^^7^ M		[31]

^1^ LOD: The minimum concentration obtained by the minimum analysis signal that can be reasonably detected by the specific analysis step; ^2^ Linear range: The range of the concentration (or amount) of the analyte corresponding to the linear portion of the calibration curve which can be measured by a specific method.

**Table 2:** Studies of SERS-based aptasensors.

Target Analyte		Matrix	LOD	Ref.
metal ion	Pb^2+^	Water	20 nM	[62]
	As^3+^	Water	59 ppt	[63]
	As^3+^	Lake water	0.1 ppb	[64]
	Hg^2+^	Water	5 nM	[65]
	Hg^2+^	Lake water	100 fM	[66]
	Hg^2+^	River water	2.5 nM	[67]
Small molecules	profenofos	Apple juice	5 ppm	[69]
	phorate	Apple juice	0.1 ppm	[69]
	phorate	Apple skin	0.05 mg/L	[85]
	omethoate	Apple juice	5 ppm	[69]
	isocarbophos	Apple juice	1 ppm	[69]
	ATP	Water	10 μM	[70]
	AFB1	Peanut milk	0.48 pg/mL	[72]
	AFB1	Peanut	0.4 fg/mL	[73]
biomacromolecules	PSA	Serum	5 pg/mL	[74]
	PSA	Serum	3.2 × 10^−20^ M	[75]
	Thrombin	Serum	5.7 × 10^−17^ M	[75]
	DNA	Serum	100 fM	[78]
	MiRNA	Serum	10 fM	[78]
	Mucin-1	Serum	4.3 aM	[77]
	Alpha fetoprotein	Serum	0.097 aM	[76]
	Lysozyme			[52]
living organisms	*S. typhimurium*	Pork	15 cfu/mL	[79]
	*S. aureus*	Pork	35 cfu/mL	[79]
	Hela cells			[81]
	CTC	Blood	1 cell/mL	[83]
	CTC	Blood	20 cells/mL	[84]
mapping	ATP	intracellular ATP molecules		[70]
	Lysozyme	different substrates		[52]
	membrane protein	Hela cells		[58]
	membrane protein	HepG2 cells		[81]

**Table 3:** Representative studies of MIPs-SERS methods.

Target Detector	MIPs-Sensor Type	Functional Monomer	Matrix	LOD	Ref.
Chlorpyrifos	MISPE-SERS	Methacrylic acid (MAA)	Apple juice	0.01 mg/L	[91]
Sudan dyes	MISPE-SERS	3-(trimethoxysilyl)propyl methacrylate (TPM)	chili powder	1.5 ng/g	[92]
Melamine	MISPE-SERS	MAA	Milk	0.005 mM	[93]
Chloramphenicol	MISPE-SERS	Acrylamide (AM)	Honey and milk	0.1 ppm	[94]
Alpha-tocopherol	MISPE-SERS	MAA	Vegetable oils	10 ppb	[95]
Sudan IV	Au NPs doped MIPs	MAA	Sudan derivatives		[89]
Theophylline	Ag NPs doped MIPs	MAA	Green tea drinks	3.5 μM	[97]
Metformin	MIM-SERS	MAA	Hypoglycemic agents	5 mg/mL	[104]
R6G	Core-shell	AM	Water	10^−12^ M	[106]
Bisphenol A	Core-shell	3-(triethoxysilyl)propyl isocyanate (TEPIC)	River water	0.1 mg/L	[107]
Transferrin	Core-shell	Polydopamine (PDA)	human serum	10^−8^ M	[123]
Glycoproteins	Sandwich structure	Boronic acids	human serum	13.8 ± 3.3 ng/mL	[122]
